# 7-Benzyl-3-(4-fluoro­phen­yl)-2-(pyrrol­idin-1-yl)-5,6,7,8-tetra­hydro­pyrido[4′,3′:4,5]thieno[2,3-*d*]pyrimidin-4(3*H*)-one

**DOI:** 10.1107/S1600536811030625

**Published:** 2011-08-02

**Authors:** Hong Chen, Hai-Jun Hu, Kai Yan, Qiu-Hong Dai

**Affiliations:** a College of Chemistry and Life Science, China Three Gorges University, Yichang 443002, People’s Republic of China; b Hubei Key Laboratory of Natural Products Research and Development, Yichang 443002, People’s Republic of China

## Abstract

In the title compound, C_26_H_25_FN_4_OS, the thienopyrimidine fused-ring system is close to planar (r.m.s. deviation = 0.066 Å), with a maximum deviation of 0.1243 (17) Å for the N atom adjacent to the carbonyl group. This ring system forms dihedral angles of 67.5 (1) and 88.9 (1) ° with the adjacent six-membered rings. Inter­molecular C—H⋯O hydrogen bonding and C—H⋯π inter­actions help to stabilize the crystal structure.

## Related literature

For the biological and pharmaceutical properties of compounds containing the fused thienopyrimidine system, see: Amr *et al.* (2010 ▶); Huang *et al.* (2009 ▶); Jennings *et al.* (2005 ▶); Kikuchi *et al.* (2006 ▶); Mavrova *et al.* (2010 ▶); Santagati *et al.* (2002 ▶). For related structures, see: Hu *et al.* (2007 ▶); Xie *et al.* (2008 ▶).
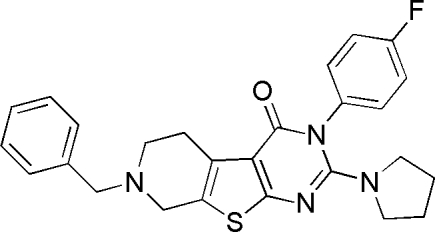


## Experimental

### 

#### Crystal data

C_26_H_25_FN_4_OS
*M* *_r_* = 460.56
Triclinic, 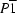

*a* = 8.132 (10) Å
*b* = 9.736 (11) Å
*c* = 15.540 (18) Å
α = 99.742 (16)°
β = 99.636 (11)°
γ = 105.551 (14)°
*V* = 1139 (2) Å^3^
*Z* = 2
Mo *K*α radiation
μ = 0.18 mm^−1^
*T* = 296 K
0.23 × 0.20 × 0.15 mm

#### Data collection

Bruker SMART CCD diffractometer
Absorption correction: multi-scan (*SADABS*; Sheldrick, 1996 ▶) *T*_min_ = 0.960, *T*_max_ = 0.974
12210 measured reflections
5233 independent reflections
4018 reflections with *I* > 2σ(*I*)
*R*_int_ = 0.154

#### Refinement

*R*[*F* ^2^ > 2σ(*F* ^2^)] = 0.064
*wR*(*F* ^2^) = 0.180
*S* = 1.05
5233 reflections
298 parameters
H-atom parameters constrained
Δρ_max_ = 0.48 e Å^−3^
Δρ_min_ = −0.38 e Å^−3^

### 

Data collection: *SMART* (Bruker, 1997 ▶); cell refinement: *SAINT* (Bruker, 1997 ▶); data reduction: *SAINT*; program(s) used to solve structure: *SHELXTL* (Sheldrick, 2008 ▶); program(s) used to refine structure: *SHELXTL*; molecular graphics: *SHELXTL*; software used to prepare material for publication: *SHELXTL*.

## Supplementary Material

Crystal structure: contains datablock(s) I, global. DOI: 10.1107/S1600536811030625/zq2114sup1.cif

Structure factors: contains datablock(s) I. DOI: 10.1107/S1600536811030625/zq2114Isup2.hkl

Supplementary material file. DOI: 10.1107/S1600536811030625/zq2114Isup3.cml

Additional supplementary materials:  crystallographic information; 3D view; checkCIF report

## Figures and Tables

**Table 1:** Hydrogen-bond geometry (Å, °) *Cg*1 and *Cg*2 are the centroids of the S1-C11-C10-C13-C16 and N2-C15-N3-C14-C13-C16 rings, respectively.

*D*—H⋯*A*	*D*—H	H⋯*A*	*D*⋯*A*	*D*—H⋯*A*
C8—H8*B*⋯O1^i^	0.97	2.50	3.461 (5)	171
C24—H24*B*⋯O1^ii^	0.97	2.44	3.316 (5)	151
C25—H25*B*⋯*Cg*1^iii^	0.97	2.86	3.693 (5)	144
C26—H26*B*⋯*Cg*2^iii^	0.97	2.80	3.717 (5)	158

Symmetry codes: (i) 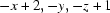; (ii) 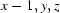; (iii) 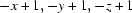.

**Fig. 1.**
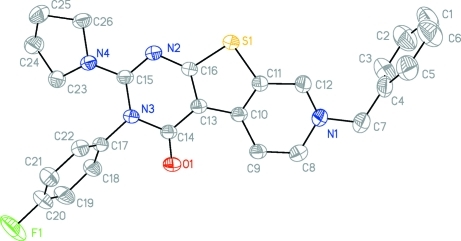
Molecular structure of the title compound with 50% probability displacement ellipsoids. H atoms are omitted for clarity.

**Fig. 2.**
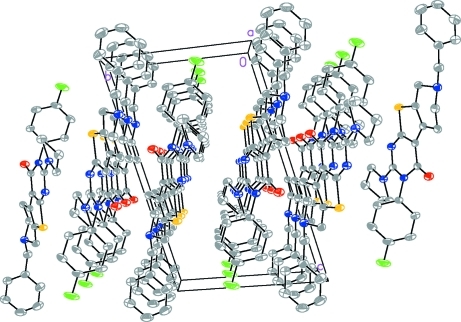
Packing diagram of the title compound projected along the a axis direction. H atoms are omitted for clarity.

**Fig. 3.**
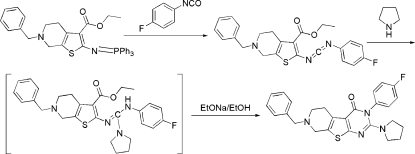
Reaction scheme.

## supplementary crystallographic information

### Comment

Derivatives of heterocycles containing the thienopyrimidine system have proved to show significant antifungal, antibacterical, anticonvulsant and angiotensin antagonistic activities (Amr *et al.* 2010; Huang *et al.* 2009; Jennings *et al.* 2005; Kikuchi *et al.* 2006; Mavrova *et al.* 2010; Santagati *et al.* 2002). Recently, we have focused on the synthesis of fused heterocyclic systems containing thienopyrimidine *via* aza-Wittig reaction under mild conditions. Some X-ray crystal structures of fused pyrimidinone derivatives have been reported (Hu *et al.*, 2007; Xie *et al.*, 2008). The title compound has potential use as a precursor for obtaining bioactive molecules with fluorescence properties. Herein, we report its crystal structure (Fig. 1 and 2).

In the crystal structure of the title compound, C_26_H_25_FN_4_OS, the thienopyrimidine fused ring system are essentially coplanar (rms deviation = 0.066 Å) with a maximum deviation of 0.1243 (17) Å for atom N3. This ring system forms dihedral angles of 67.5 (1) and 88.9 (1) ° with the adjacent 6-membered rings C17–C22 and C1-C6, respectively. Intermolecular C—H···O hydrogen bondings (C8—H8B···O1^i^ and C24—H24B···O1^ii^ with symmetry codes: (i) -*x*+2, -*y*, -*z*+1; (ii) *x*-1, *y*, *z*) and C—H···π interactions (C25—H25B···Cg1^iii^ and C26—H26···Cg2^iii^ with Cg1 and Cg2 centroids of the S1-C11-C10-C13-C16 and N2-C15-N3-C14-C13-C16 rings and symmetry code: (iii) 1-x, 1-y, 1-z) help to stabilize the crystal structure of the title compound (Table 1).

### Experimental

Fluoro-4-isocyanatobenzene (2 mmol) under nitrogen atmosphere was added to a solution of iminophosphorane (2 mmol) in anhydrous CH_2_Cl_2_ (10 ml) at room temperature (Fig. 3). When the reaction mixture was left unstirred for 12 h at 273–278k, iminophosphorane was consumed (TLC monitored). The solvent was removed under reduced pressure and ether/petroleum ether (volume ratio 1:2, 20 ml) was added to precipitate triphenylphosphine oxide. Removal of the solvent gave carbodiimide, which was used directly without further purification. Pyrrolidine (2 mmol) was added to the solution of carbodiimide in anhydrous dichloromethane (10 ml). After the reaction mixture was left unstirred for 5–6 h, the solvent was removed and anhydrous EtOH (10 ml) with several drops of EtONa (in EtOH) was added to the mixture. The mixture was stirred for another 6–8 h at room temperature. The solution was condensed and the residual was recrystallized from EtOH to give the expected title compound as white crystals.

### Refinement

All H atoms were positioned geometrically [C—H = 0.93, 0.97 Å] and allowed to ride on their parent atoms, with *U*_iso_(H) = 1.2*U*_eq_(C).

### Crystal data

**Table:**

C_26_H_25_FN_4_OS	*Z* = 2
*M**_r_* = 460.56	*F*(000) = 484
Triclinic, *P*1	*D*_x_ = 1.343 Mg m^−^^3^
Hall symbol: -P 1	Melting point: 485 K
*a* = 8.132 (10) Å	Mo *K*α radiation, λ = 0.71073 Å
*b* = 9.736 (11) Å	Cell parameters from 2106 reflections
*c* = 15.540 (18) Å	θ = 2.7–27.5°
α = 99.742 (16)°	µ = 0.18 mm^−^^1^
β = 99.636 (11)°	*T* = 296 K
γ = 105.551 (14)°	Block, colourless
*V* = 1139 (2) Å^3^	0.23 × 0.20 × 0.15 mm

### Data collection

**Table:**

Bruker SMART CCD diffractometer	5233 independent reflections
Radiation source: fine-focus sealed tube	4018 reflections with *I* > 2σ(*I*)
graphite	*R*_int_ = 0.154
CCD Profile fitting scans	θ_max_ = 27.5°, θ_min_ = 2.7°
Absorption correction: multi-scan (*SADABS*; Sheldrick, 1996)	*h* = −10→10
*T*_min_ = 0.960, *T*_max_ = 0.974	*k* = −12→12
12210 measured reflections	*l* = −20→20

### Refinement

**Table:**

Refinement on *F*^2^	Primary atom site location: structure-invariant direct methods
Least-squares matrix: full	Secondary atom site location: difference Fourier map
*R*[*F*^2^ > 2σ(*F*^2^)] = 0.064	Hydrogen site location: inferred from neighbouring sites
*wR*(*F*^2^) = 0.180	H-atom parameters constrained
*S* = 1.05	*w* = 1/[σ^2^(*F*_o_^2^) + (0.0691*P*)^2^] where *P* = (*F*_o_^2^ + 2*F*_c_^2^)/3
5233 reflections	(Δ/σ)_max_ < 0.001
298 parameters	Δρ_max_ = 0.48 e Å^−^^3^
0 restraints	Δρ_min_ = −0.38 e Å^−^^3^

### Special details

**Table:**

Geometry. All e.s.d.'s (except the e.s.d. in the dihedral angle between two l.s. planes) are estimated using the full covariance matrix. The cell e.s.d.'s are taken into account individually in the estimation of e.s.d.'s in distances, angles and torsion angles; correlations between e.s.d.'s in cell parameters are only used when they are defined by crystal symmetry. An approximate (isotropic) treatment of cell e.s.d.'s is used for estimating e.s.d.'s involving l.s. planes.
Refinement. Refinement of *F*^2^ against ALL reflections. The weighted *R*-factor *wR* and goodness of fit *S* are based on *F*^2^, conventional *R*-factors *R* are based on *F*, with *F* set to zero for negative *F*^2^. The threshold expression of *F*^2^ > σ(*F*^2^) is used only for calculating *R*-factors(gt) *etc*. and is not relevant to the choice of reflections for refinement. *R*-factors based on *F*^2^ are statistically about twice as large as those based on *F*, and *R*- factors based on ALL data will be even larger.

### Fractional atomic coordinates and isotropic or equivalent isotropic displacement parameters (Å^2^)

**Table:**

	*x*	*y*	*z*	*U*_iso_*/*U*_eq_	
S1	0.44564 (6)	0.17251 (6)	0.31146 (3)	0.04008 (18)	
N2	0.3853 (2)	0.28656 (17)	0.47059 (11)	0.0354 (4)	
O1	0.7783 (2)	0.16116 (16)	0.60615 (10)	0.0477 (4)	
N3	0.57983 (19)	0.29116 (16)	0.60532 (10)	0.0318 (3)	
N4	0.3472 (2)	0.39607 (17)	0.60525 (11)	0.0349 (4)	
C13	0.6094 (2)	0.16139 (18)	0.46627 (13)	0.0322 (4)	
C15	0.4366 (2)	0.32359 (19)	0.55846 (13)	0.0315 (4)	
C17	0.6746 (2)	0.3752 (2)	0.69460 (12)	0.0335 (4)	
C14	0.6647 (2)	0.19681 (19)	0.56157 (13)	0.0328 (4)	
C16	0.4790 (2)	0.21316 (19)	0.42757 (13)	0.0332 (4)	
N1	0.7929 (2)	−0.04640 (17)	0.25491 (12)	0.0401 (4)	
C10	0.6883 (2)	0.09292 (18)	0.40137 (13)	0.0329 (4)	
C23	0.3336 (3)	0.4011 (2)	0.69915 (14)	0.0403 (4)	
H23A	0.4258	0.4826	0.7396	0.048*	
H23B	0.3403	0.3110	0.7161	0.048*	
C11	0.6146 (3)	0.0929 (2)	0.31656 (13)	0.0351 (4)	
C22	0.7449 (3)	0.5255 (2)	0.70756 (14)	0.0393 (4)	
H22	0.7291	0.5706	0.6600	0.047*	
C26	0.2043 (2)	0.4368 (2)	0.55417 (15)	0.0402 (5)	
H26A	0.1182	0.3514	0.5136	0.048*	
H26B	0.2495	0.5072	0.5200	0.048*	
C9	0.8400 (3)	0.0331 (2)	0.41996 (14)	0.0381 (4)	
H9A	0.7991	−0.0636	0.4318	0.046*	
H9B	0.9257	0.0961	0.4726	0.046*	
C12	0.6811 (3)	0.0451 (2)	0.23597 (14)	0.0399 (5)	
H12A	0.5828	−0.0095	0.1865	0.048*	
H12B	0.7473	0.1305	0.2184	0.048*	
C18	0.7011 (3)	0.3054 (2)	0.76407 (14)	0.0452 (5)	
H18	0.6543	0.2044	0.7545	0.054*	
C8	0.9246 (3)	0.0243 (2)	0.33961 (15)	0.0435 (5)	
H8A	0.9891	0.1222	0.3367	0.052*	
H8B	1.0071	−0.0308	0.3473	0.052*	
F1	0.9572 (3)	0.6188 (2)	0.94182 (12)	0.1047 (7)	
C4	0.7603 (3)	−0.1314 (3)	0.09123 (16)	0.0513 (6)	
C21	0.8389 (3)	0.6084 (3)	0.79164 (17)	0.0530 (6)	
H21	0.8838	0.7097	0.8019	0.064*	
C19	0.7981 (3)	0.3875 (3)	0.84799 (16)	0.0596 (6)	
H19	0.8183	0.3429	0.8954	0.072*	
C24	0.1546 (3)	0.4208 (3)	0.70031 (16)	0.0516 (6)	
H24A	0.1543	0.4772	0.7580	0.062*	
H24B	0.0648	0.3269	0.6874	0.062*	
C25	0.1256 (3)	0.5036 (2)	0.62624 (17)	0.0504 (5)	
H25A	0.0018	0.4885	0.6040	0.061*	
H25B	0.1850	0.6077	0.6478	0.061*	
C7	0.8822 (3)	−0.0742 (3)	0.18182 (17)	0.0520 (6)	
H7A	0.9434	−0.1444	0.1933	0.062*	
H7B	0.9690	0.0161	0.1816	0.062*	
C20	0.8637 (3)	0.5372 (3)	0.85920 (16)	0.0621 (7)	
C3	0.6252 (4)	−0.2613 (3)	0.0722 (2)	0.0719 (8)	
H3	0.6104	−0.3154	0.1158	0.086*	
C5	0.7773 (4)	−0.0546 (3)	0.0246 (2)	0.0709 (8)	
H5	0.8661	0.0339	0.0366	0.085*	
C6	0.6658 (5)	−0.1054 (4)	−0.0599 (2)	0.0875 (10)	
H6	0.6812	−0.0521	−0.1039	0.105*	
C2	0.5115 (5)	−0.3116 (4)	−0.0117 (2)	0.0837 (9)	
H2	0.4197	−0.3982	−0.0237	0.100*	
C1	0.5346 (6)	−0.2330 (4)	−0.0777 (2)	0.0861 (10)	
H1	0.4599	−0.2680	−0.1342	0.103*	

### Atomic displacement parameters (Å^2^)

**Table:**

	*U*^11^	*U*^22^	*U*^33^	*U*^12^	*U*^13^	*U*^23^
S1	0.0408 (3)	0.0565 (3)	0.0278 (3)	0.0268 (2)	0.0029 (2)	0.0076 (2)
N2	0.0305 (8)	0.0460 (9)	0.0319 (8)	0.0176 (7)	0.0042 (6)	0.0075 (7)
O1	0.0476 (9)	0.0631 (9)	0.0363 (8)	0.0338 (7)	−0.0036 (6)	0.0069 (7)
N3	0.0275 (7)	0.0394 (8)	0.0274 (8)	0.0124 (6)	0.0015 (6)	0.0056 (7)
N4	0.0297 (8)	0.0465 (8)	0.0312 (8)	0.0171 (7)	0.0059 (6)	0.0080 (7)
C13	0.0308 (9)	0.0346 (9)	0.0324 (10)	0.0131 (7)	0.0039 (7)	0.0082 (8)
C15	0.0261 (8)	0.0378 (9)	0.0307 (9)	0.0104 (7)	0.0040 (7)	0.0092 (8)
C17	0.0274 (8)	0.0467 (10)	0.0265 (9)	0.0141 (8)	0.0035 (7)	0.0062 (8)
C14	0.0301 (9)	0.0346 (9)	0.0326 (10)	0.0120 (7)	0.0015 (7)	0.0065 (8)
C16	0.0301 (9)	0.0403 (9)	0.0284 (9)	0.0140 (7)	0.0015 (7)	0.0058 (8)
N1	0.0432 (9)	0.0462 (9)	0.0363 (9)	0.0233 (8)	0.0103 (7)	0.0063 (8)
C10	0.0313 (9)	0.0341 (8)	0.0330 (10)	0.0128 (7)	0.0027 (7)	0.0065 (8)
C23	0.0353 (10)	0.0537 (11)	0.0334 (10)	0.0143 (9)	0.0110 (8)	0.0093 (9)
C11	0.0377 (10)	0.0399 (9)	0.0307 (10)	0.0183 (8)	0.0056 (8)	0.0076 (8)
C22	0.0326 (9)	0.0475 (10)	0.0375 (11)	0.0147 (8)	0.0062 (8)	0.0064 (9)
C26	0.0283 (9)	0.0514 (11)	0.0429 (12)	0.0166 (8)	0.0051 (8)	0.0119 (9)
C9	0.0352 (10)	0.0406 (9)	0.0371 (10)	0.0169 (8)	0.0003 (8)	0.0041 (8)
C12	0.0457 (11)	0.0466 (10)	0.0332 (10)	0.0233 (9)	0.0094 (9)	0.0090 (9)
C18	0.0461 (12)	0.0581 (12)	0.0318 (11)	0.0178 (10)	0.0051 (9)	0.0117 (10)
C8	0.0380 (11)	0.0500 (11)	0.0460 (12)	0.0208 (9)	0.0074 (9)	0.0097 (10)
F1	0.1166 (17)	0.1151 (14)	0.0450 (10)	0.0321 (12)	−0.0342 (10)	−0.0244 (10)
C4	0.0655 (15)	0.0610 (13)	0.0401 (12)	0.0356 (12)	0.0211 (11)	0.0099 (11)
C21	0.0433 (12)	0.0515 (12)	0.0502 (14)	0.0117 (10)	−0.0017 (10)	−0.0087 (11)
C19	0.0610 (15)	0.0860 (18)	0.0301 (11)	0.0277 (13)	−0.0020 (10)	0.0119 (12)
C24	0.0349 (11)	0.0754 (15)	0.0473 (13)	0.0192 (10)	0.0149 (9)	0.0119 (12)
C25	0.0361 (11)	0.0599 (13)	0.0606 (15)	0.0225 (10)	0.0135 (10)	0.0123 (12)
C7	0.0541 (13)	0.0638 (13)	0.0486 (14)	0.0317 (11)	0.0187 (11)	0.0111 (11)
C20	0.0575 (15)	0.0801 (17)	0.0335 (12)	0.0234 (13)	−0.0117 (10)	−0.0104 (12)
C3	0.102 (2)	0.0659 (16)	0.0445 (15)	0.0245 (16)	0.0109 (14)	0.0097 (13)
C5	0.0764 (19)	0.0890 (19)	0.0595 (18)	0.0321 (16)	0.0261 (15)	0.0278 (16)
C6	0.111 (3)	0.109 (3)	0.056 (2)	0.043 (2)	0.0215 (19)	0.037 (2)
C2	0.108 (3)	0.0735 (18)	0.0561 (19)	0.0235 (17)	0.0034 (16)	−0.0004 (16)
C1	0.121 (3)	0.100 (2)	0.0404 (15)	0.053 (2)	0.0078 (17)	0.0041 (16)

### Geometric parameters (Å, °)

**Table:**

S1—C16	1.739 (3)	C9—H9B	0.9700
S1—C11	1.747 (2)	C12—H12A	0.9700
N2—C15	1.316 (3)	C12—H12B	0.9700
N2—C16	1.357 (3)	C18—C19	1.388 (3)
O1—C14	1.222 (2)	C18—H18	0.9300
N3—C15	1.406 (2)	C8—H8A	0.9700
N3—C14	1.438 (3)	C8—H8B	0.9700
N3—C17	1.452 (3)	F1—C20	1.364 (3)
N4—C15	1.354 (3)	C4—C5	1.380 (4)
N4—C23	1.475 (3)	C4—C3	1.383 (4)
N4—C26	1.478 (3)	C4—C7	1.499 (4)
C13—C16	1.384 (3)	C21—C20	1.369 (4)
C13—C14	1.427 (3)	C21—H21	0.9300
C13—C10	1.446 (3)	C19—C20	1.380 (4)
C17—C22	1.387 (3)	C19—H19	0.9300
C17—C18	1.386 (3)	C24—C25	1.535 (3)
N1—C12	1.463 (3)	C24—H24A	0.9700
N1—C7	1.473 (3)	C24—H24B	0.9700
N1—C8	1.476 (3)	C25—H25A	0.9700
C10—C11	1.353 (3)	C25—H25B	0.9700
C10—C9	1.506 (3)	C7—H7A	0.9700
C23—C24	1.521 (3)	C7—H7B	0.9700
C23—H23A	0.9700	C3—C2	1.391 (4)
C23—H23B	0.9700	C3—H3	0.9300
C11—C12	1.497 (3)	C5—C6	1.390 (5)
C22—C21	1.386 (3)	C5—H5	0.9300
C22—H22	0.9300	C6—C1	1.354 (5)
C26—C25	1.518 (3)	C6—H6	0.9300
C26—H26A	0.9700	C2—C1	1.387 (5)
C26—H26B	0.9700	C2—H2	0.9300
C9—C8	1.524 (3)	C1—H1	0.9300
C9—H9A	0.9700		
			
C16—S1—C11	91.27 (9)	C11—C12—H12B	109.5
C15—N2—C16	115.47 (16)	H12A—C12—H12B	108.1
C15—N3—C14	121.92 (17)	C19—C18—C17	119.4 (2)
C15—N3—C17	121.57 (16)	C19—C18—H18	120.3
C14—N3—C17	115.13 (15)	C17—C18—H18	120.3
C15—N4—C23	127.56 (16)	N1—C8—C9	111.67 (19)
C15—N4—C26	117.88 (18)	N1—C8—H8A	109.3
C23—N4—C26	111.67 (16)	C9—C8—H8A	109.3
C16—C13—C14	118.39 (17)	N1—C8—H8B	109.3
C16—C13—C10	112.99 (18)	C9—C8—H8B	109.3
C14—C13—C10	128.06 (17)	H8A—C8—H8B	107.9
N2—C15—N4	118.25 (17)	C5—C4—C3	117.9 (3)
N2—C15—N3	122.75 (17)	C5—C4—C7	120.9 (3)
N4—C15—N3	118.99 (18)	C3—C4—C7	121.2 (2)
C22—C17—C18	120.97 (19)	C20—C21—C22	118.2 (2)
C22—C17—N3	118.44 (17)	C20—C21—H21	120.9
C18—C17—N3	120.52 (18)	C22—C21—H21	120.9
O1—C14—C13	126.76 (18)	C20—C19—C18	118.2 (2)
O1—C14—N3	119.67 (19)	C20—C19—H19	120.9
C13—C14—N3	113.47 (15)	C18—C19—H19	120.9
N2—C16—C13	127.03 (19)	C23—C24—C25	103.86 (17)
N2—C16—S1	121.94 (14)	C23—C24—H24A	111.0
C13—C16—S1	111.04 (14)	C25—C24—H24A	111.0
C12—N1—C7	111.41 (18)	C23—C24—H24B	111.0
C12—N1—C8	110.24 (16)	C25—C24—H24B	111.0
C7—N1—C8	109.4 (2)	H24A—C24—H24B	109.0
C11—C10—C13	111.91 (18)	C26—C25—C24	103.37 (18)
C11—C10—C9	120.74 (18)	C26—C25—H25A	111.1
C13—C10—C9	127.28 (18)	C24—C25—H25A	111.1
N4—C23—C24	103.68 (16)	C26—C25—H25B	111.1
N4—C23—H23A	111.0	C24—C25—H25B	111.1
C24—C23—H23A	111.0	H25A—C25—H25B	109.1
N4—C23—H23B	111.0	N1—C7—C4	113.4 (2)
C24—C23—H23B	111.0	N1—C7—H7A	108.9
H23A—C23—H23B	109.0	C4—C7—H7A	108.9
C10—C11—C12	124.32 (19)	N1—C7—H7B	108.9
C10—C11—S1	112.73 (15)	C4—C7—H7B	108.9
C12—C11—S1	122.64 (15)	H7A—C7—H7B	107.7
C21—C22—C17	119.8 (2)	F1—C20—C21	118.2 (3)
C21—C22—H22	120.1	F1—C20—C19	118.5 (3)
C17—C22—H22	120.1	C21—C20—C19	123.3 (2)
N4—C26—C25	103.64 (19)	C4—C3—C2	120.4 (3)
N4—C26—H26A	111.0	C4—C3—H3	119.8
C25—C26—H26A	111.0	C2—C3—H3	119.8
N4—C26—H26B	111.0	C4—C5—C6	122.0 (3)
C25—C26—H26B	111.0	C4—C5—H5	119.0
H26A—C26—H26B	109.0	C6—C5—H5	119.0
C10—C9—C8	109.86 (18)	C1—C6—C5	119.5 (3)
C10—C9—H9A	109.7	C1—C6—H6	120.3
C8—C9—H9A	109.7	C5—C6—H6	120.3
C10—C9—H9B	109.7	C1—C2—C3	120.2 (4)
C8—C9—H9B	109.7	C1—C2—H2	119.9
H9A—C9—H9B	108.2	C3—C2—H2	119.9
N1—C12—C11	110.81 (17)	C6—C1—C2	120.1 (3)
N1—C12—H12A	109.5	C6—C1—H1	120.0
C11—C12—H12A	109.5	C2—C1—H1	120.0
N1—C12—H12B	109.5		
			
C16—N2—C15—N4	−179.48 (15)	C9—C10—C11—S1	−178.14 (13)
C16—N2—C15—N3	0.6 (3)	C16—S1—C11—C10	1.92 (16)
C23—N4—C15—N2	155.38 (18)	C16—S1—C11—C12	−172.02 (17)
C26—N4—C15—N2	−3.6 (2)	C18—C17—C22—C21	−1.9 (3)
C23—N4—C15—N3	−24.7 (3)	N3—C17—C22—C21	−178.84 (18)
C26—N4—C15—N3	176.30 (15)	C15—N4—C26—C25	175.64 (16)
C14—N3—C15—N2	−9.2 (3)	C23—N4—C26—C25	13.4 (2)
C17—N3—C15—N2	156.74 (18)	C11—C10—C9—C8	18.0 (2)
C14—N3—C15—N4	170.88 (16)	C13—C10—C9—C8	−158.70 (18)
C17—N3—C15—N4	−23.2 (2)	C7—N1—C12—C11	−170.65 (17)
C15—N3—C17—C22	−55.7 (2)	C8—N1—C12—C11	−49.0 (2)
C14—N3—C17—C22	111.1 (2)	C10—C11—C12—N1	19.6 (3)
C15—N3—C17—C18	127.3 (2)	S1—C11—C12—N1	−167.21 (13)
C14—N3—C17—C18	−65.8 (2)	C22—C17—C18—C19	0.6 (3)
C16—C13—C14—O1	−179.38 (18)	N3—C17—C18—C19	177.5 (2)
C10—C13—C14—O1	−8.6 (3)	C12—N1—C8—C9	66.5 (2)
C16—C13—C14—N3	−3.1 (2)	C7—N1—C8—C9	−170.69 (17)
C10—C13—C14—N3	167.70 (16)	C10—C9—C8—N1	−48.3 (2)
C15—N3—C14—O1	−173.49 (17)	C17—C22—C21—C20	2.0 (3)
C17—N3—C14—O1	19.7 (2)	C17—C18—C19—C20	0.5 (4)
C15—N3—C14—C13	9.9 (2)	N4—C23—C24—C25	−29.0 (2)
C17—N3—C14—C13	−156.85 (16)	N4—C26—C25—C24	−31.0 (2)
C15—N2—C16—C13	6.9 (3)	C23—C24—C25—C26	37.6 (2)
C15—N2—C16—S1	−173.02 (14)	C12—N1—C7—C4	−51.8 (3)
C14—C13—C16—N2	−5.5 (3)	C8—N1—C7—C4	−173.91 (18)
C10—C13—C16—N2	−177.61 (17)	C5—C4—C7—N1	118.8 (3)
C14—C13—C16—S1	174.45 (14)	C3—C4—C7—N1	−60.0 (3)
C10—C13—C16—S1	2.31 (19)	C22—C21—C20—F1	179.6 (2)
C11—S1—C16—N2	177.55 (16)	C22—C21—C20—C19	−0.8 (4)
C11—S1—C16—C13	−2.38 (14)	C18—C19—C20—F1	179.2 (2)
C16—C13—C10—C11	−0.9 (2)	C18—C19—C20—C21	−0.4 (4)
C14—C13—C10—C11	−172.11 (18)	C5—C4—C3—C2	0.1 (4)
C16—C13—C10—C9	176.07 (17)	C7—C4—C3—C2	178.9 (3)
C14—C13—C10—C9	4.9 (3)	C3—C4—C5—C6	−1.2 (4)
C15—N4—C23—C24	−150.15 (19)	C7—C4—C5—C6	180.0 (3)
C26—N4—C23—C24	9.9 (2)	C4—C5—C6—C1	0.9 (5)
C13—C10—C11—C12	172.87 (17)	C4—C3—C2—C1	1.2 (5)
C9—C10—C11—C12	−4.3 (3)	C5—C6—C1—C2	0.4 (5)
C13—C10—C11—S1	−0.9 (2)	C3—C2—C1—C6	−1.4 (5)

### Hydrogen-bond geometry (Å, °)

**Table:**

Cg1 and Cg2 are the centroids of the S1-C11-C10-C13-C16 and N2-C15-N3-C14-C13-C16 rings, respectively.

**Table:**

*D*—H···*A*	*D*—H	H···*A*	*D*···*A*	*D*—H···*A*
C8—H8B···O1^i^	0.97	2.50	3.461 (5)	171
C24—H24B···O1^ii^	0.97	2.44	3.316 (5)	151
C25—H25B···Cg1^iii^	0.97	2.86	3.693 (5)	144
C26—H26B···Cg2^iii^	0.97	2.80	3.717 (5)	158

Symmetry codes: (i) −*x*+2, −*y*, −*z*+1; (ii) *x*−1, *y*, *z*; (iii) −*x*+1, −*y*+1, −*z*+1.
